# A prospective matched study on symptomatic dengue in pregnancy

**DOI:** 10.1371/journal.pone.0202005

**Published:** 2018-10-03

**Authors:** Célia Basurko, Sibille Everhard, Séverine Matheus, Marion Restrepo, Hélène Hildéral, Véronique Lambert, Rachida Boukhari, Jean-Pierre Duvernois, Anne Favre, Larissa Valmy, Mathieu Nacher, Gabriel Carles

**Affiliations:** 1 Centre d'Investigation Clinique Antilles Guyane, Inserm CIC1424, Centre Hospitalier de Cayenne, Cayenne, French Guiana, France; 2 Equipe EA3593, Ecosystèmes amazoniens et Pathologie Tropicale, Université de la Guyane, Cayenne, French Guiana, France; 3 Laboratoire de virologie, Centre National de Référence des Arbovirus, Institut Pasteur de la Guyane Cayenne, French Guiana, France; 4 Service de gynécologie et d'obstétrique, Centre Hospitalier de l'Ouest Guyanais, Saint Laurent du Maroni, French Guiana, France; 5 Pôle de santé publique, Centre Hospitalier de l'Ouest Guyanais, Saint Laurent du Maroni, French Guiana, France; 6 Service d'analyses médicales, Centre Hospitalier de l'Ouest Guyanais, Saint Laurent du Maroni, French Guiana, France; 7 Service de gynécologie et d'obstétrique, Centre médico-chirurgical de Kourou, Croix Rouge Française, Kourou, French Guiana, France; 8 Réseau périnat de Guyane, Cayenne, French Guiana, France; CEA, FRANCE

## Abstract

Dengue fever is an increasing problem worldwide, but consequences during pregnancy remain unclear. Much of the available literature suffers from methodological biases that compromise the validity of clinical recommendations. We conducted a matched cohort study during an epidemic in French Guiana to compare events and pregnancy outcomes between two paired groups of pregnant women: women having presented with symptomatic dengue during pregnancy (n = 73) and women having had neither fever nor dengue during pregnancy (n = 219). Women in each arm were matched by place of follow up, gestation weeks at inclusion, and place of residence. Dengue infection was considered to be confirmed if viral RNA, N S1 antigen, the seroconversion of IgM antibodies or the presence of IgM was detected in collected samples. According to the 2009 WHO classification, 27% of the women with symptomatic dengue had at least one clinical or biological warning sign. These complications occurred after the 28th week of gestation in 55% of cases. The medical history, socioeconomic status and demographic characteristics were included in multivariate analysis. Exposure to dengue during pregnancy was not significantly associated with prematurity, small for gestational age infants, hypertension or emergency caesarian section. Maternal dengue with warning signs was a risk factor for peripartum hemorrhage with adjusted relative risk = 8.6(95% CI = 1.2–62). There was a near significant association between dengue and *in utero* death (p = 0.09). This prospective comparative study underlined the importance of taking into account potential confounders between exposure to dengue and the occurrence of obstetrical events. It also confirms the need for increased vigilance for pregnant women with dengue, particularly for women who present with severe dengue.

## Introduction

Although the incidence of dengue was multiplied by 30 over the last 50 years, few rigorous investigations of the consequences of this disease during pregnancy have been published. There is a lack of comparative studies and the studies reported (mostly case reports and case series) are prone to bias due to the retrospective nature of data collection, the definition criteria for acute dengue, and the inadequate adjustment for potential confounders notably obstetrical history [1,2]. The main obstetrical pathologies reported during dengue in pregnant women are premature labor, a high risk of miscarriage, low birth weight and deliverance hemorrhage [1,3–6]. The new WHO guidelines call for vigilance in cases of dengue during pregnancy, but it is still unclear whether dengue has significant effects on the course and the outcome of pregnancy [7]. In 2012–2013, a dengue epidemic struck French Guiana: it was estimated that 13,240 people were infected (5.7% of the population), 4% presented dengue with warning signs, and the severity rate was 0.5% according to the WHO’s 2009 classification. During this epidemic, there were five deaths attributable to the dengue virus, and four of them were women. The major agent (95% of cases) was dengue virus serotype 2 (DENV-2), although other serotypes cocirculated (DENV-4 < 5%; DENV-1 and DENV-3 < 1% each).

We conducted a multicentric comparative study in French Guiana during the most recent epidemic to determine the rate of pathological outcomes of pregnancy for women with symptomatic dengue by comparaison to women not infected with dengue virus. Here, we present the results of this matched cohort study on dengue during pregnancy.

## Materials and methods

### Study design and setting

**Fig 1:**
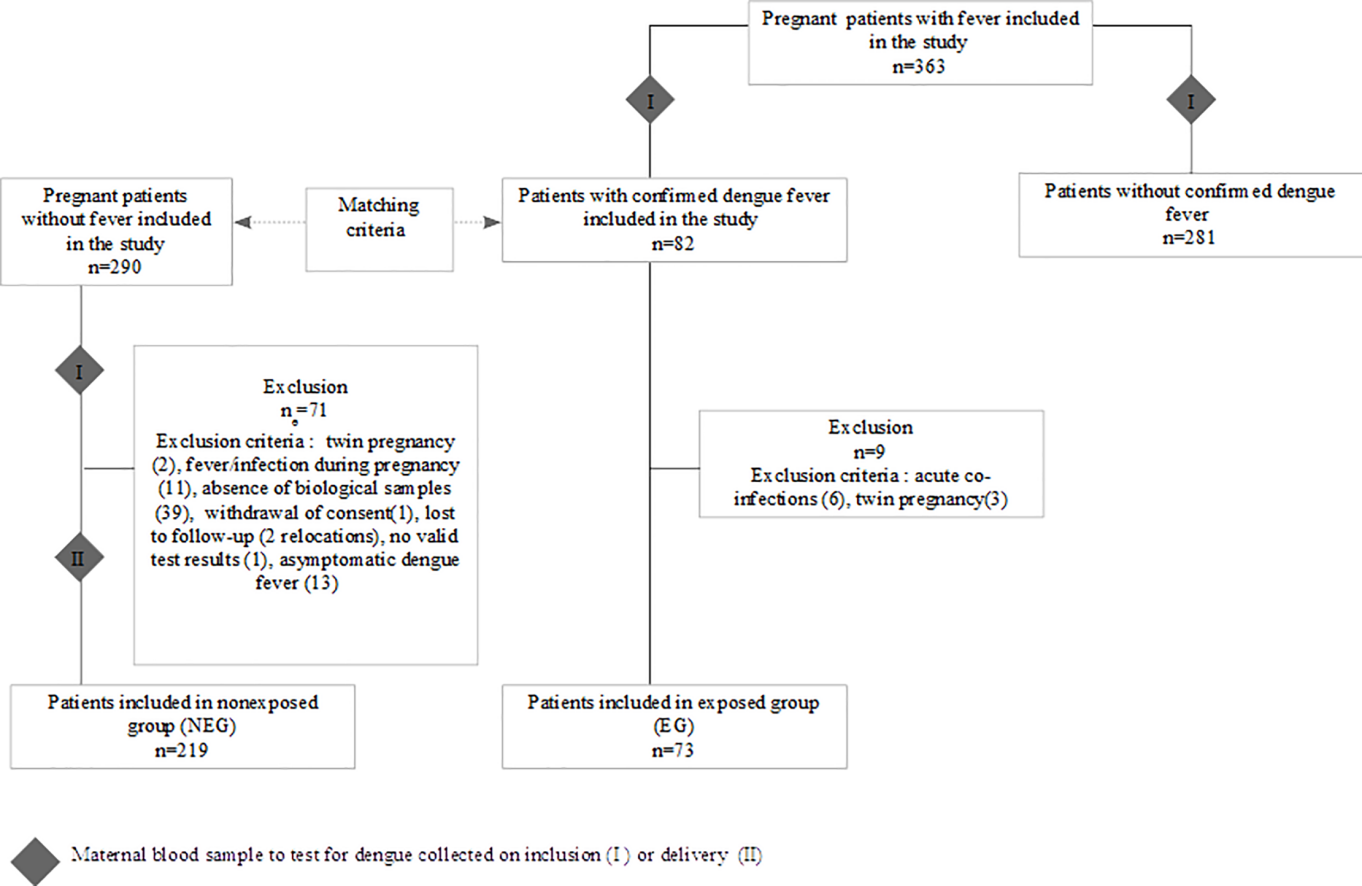
Study flow chart.

The study was a prospective multicentric matched cohort study (Fig 1).

It was proposed to all women presenting with fever and/or a suspicion of dengue during pregnancy (based on the presence of headaches, retro-orbital pain, muscle pain, joint pain, or rash) followed in one of the centers participating in the study (hospitals, mother and child care centers, and private practitioners in French Guiana). After obtaining signed informed consent, the participant gave a peripheral blood sample used to test for the dengue virus. In the cases where dengue fever was laboratory-confirmed the participant was included in the "exposed" group (EG) at the time of the symptomatic dengue episode. In other cases, she was excluded from the study. For each new exposed woman (as soon as the dengue was confirmed by the laboratory), three pregnant women without fever were recruited to constitute a control group of "non exposed group" (NEG). The three eligible women consulting immediately following the inclusion of an exposed woman in the same center were used as the unexposed matches for the exposed women. After these NEG women signed the informed consent, a peripheral blood sample was taken to test for the dengue virus, and any woman with biological signs of a recent infection was excluded.

Both groups of women were followed until delivery and the immediate postpartum period (generally between 2 and 5 days after delivery). The study was observational and the normal follow up of pregnancy (frequency and nature of clinical and paraclinical examinations) was not modified for the study.

### Matching criteria

Women of NEG and those from the EG were matched by place of follow up (hospital, mother and child care center, or private practitioner), term at inclusion (<14 weeks of gestation WG and > = 14 WG), and place of residence (Cayenne, central part of Guiana, western Guiana). These three matching criteria were chosen to minimize both measurement and selection biases. Socio-economic status, lifestyle and obstetric history in French Guiana differ according to the place of residence and the medical monitoring center.

### Biological analysis

Immediately after participants (EG and NEG) had signed the informed consent form (the time of inclusion), they gave a peripheral blood sample, which was tested for dengue. Women in the NEG group gave another blood sample at the time of delivery to test for dengue infection, in case the women were subsequently infected between inclusion and delivery.

Biological diagnosis of dengue infection was performed by the French National Reference Center for arboviruses in Cayenne. Molecular and serological investigations performed depended on the specimen volume available: detection of viral RNA genome by reverse transcriptase-polymerase chain reaction (RT-PCR) as described by Lanciotti *et al*.[8], detection of non structural (NS)1 Antigen by the *Platelia*^TM^ Dengue NS1 Ag capture ELISA (Bio-Rad laboratories, Marnes-La-Coquette, France)[9] and testing for specific dengue IgM antibodies by MAC using an “in-house” protocol described by Talarmin *et al* [10].

Previous exposure of the patients to dengue virus was determined using Panbio Dengue IgM capture ELISA (Cat. No. E-DEN01M) and Dengue IgG capture ELISA (Cat. No. E-DEN02G) according to the manufacturer's instructions (Panbio, Brisbane, Australia). The dengue virus E and M protein-specific IgM:IgG ratio was used to distinguish primary from secondary infections. According to this method, the dengue infection was defined as a primary infection if the IgM:IgG ratio was greater than 1.2 (1:100 dilution), or as a secondary infection if the ratio was less than 1.2.

All patients included in the EG had negative IgM antibodies directed against other arboviruses routinely tested by the National Reference Center of Arboviruses (Institut Pasteur de la Guyane) (Yellow Fever, Saint Louis Encephalitis, Tonate virus, Mayaro and Chikungunya virus).

### Definition of groups and exposure

To be included in the exposed group (EG) women had to have laboratory-confirmed symptomatic dengue during pregnancy. Dengue infection (exposure) was considered to be confirmed if viral RNA, NS1 antigen, the seroconversion of IgM antibodies or the presence of IgM was detected in collected samples.

To be included in the non exposed group (NEG) women had to have no dengue infection and no signs of fever (over 38.5°C for more than 48h) at any time during pregnancy. Pregnant women were considered to be non exposed to the dengue virus (NEG) if all NS1 antigen tests and specific dengue IgM tests on blood samples between inclusion and delivery were negative. Only pregnant women with none of the non-inclusion criteria and with the inclusion criteria were included in the study. Any participant presenting with any of the exclusion criteria after inclusion was excluded from the study (Table 1).

**Table 1:** Non inclusion criteria and exclusion criteria in the the study (for NEG and EG).

**Non inclusion criteria** ^**1**^	**Description and measure**
Acute co-infection	Chikungunya: positive RT-PCR or positive specific IgM
	Malaria: parasites detected in blood smears
	HIV primo-infection: positive serology
	Toxoplasmosis: seroconversion during pregnancy (IgM/IgG serology)
	Rubella: seroconversion during pregnancy (IgM/IgG serology)
	Chicken pox: seroconversion during pregnancy (IgM/IgG serology)
	CMV infection: seroconversion during pregnancy (IgM/IgG serology)
	Listeriosis: positive blood culture
Twin pregnancy	Determined by ultrasound
Medically assisted pregnancy	Interview/medical staff
Preexisting gynecological pathologies	Sub mucous fibroma, operated pelvic static problems, genital cancer, genital malformation, *in utero* exposure to distilbene
**Exclusion criteria** ^**2**^	**Description and measure**
Fever	Over 38.5°C for more than 48h
Lost to follow-up	Relocation for delivery
Breach of protocol	No biological sample available for inclusion and/or delivery, withdrawal of consent, inclusion not valid, no valid biological test results
Dengue (NEG only)	RT-PCR, NS1 or IgM results positive

^1^ Non Inclusion criteria are characteristics that the subjects did not must have if they were to be included in the study

^2^ Exclusion criteria are characteristics that exclude subjects from the study in progress

### Data collection

Data were collected from medical records, and at the patient’s bedside (data about the standard of care). Socio economic data were collected using a standardized questionnaire with 11 items and used to calculate a multidimensional social vulnerability score (score EPICES[11,12]). This score reflects social and material difficulties, and social isolation. A score above 30.17 was considered to indicate vulnerability.

### Outcome definitions (Table 2)

Gestational age was determined by ultrasound.

**Table 2:** Outcome definitions in the the study.

Outcomes	Definitions and mesures
Premature delivery	Delivery before 37 weeks of gestation (WG) and after 22 WG of a live newborn weighing more than 500 grams, excluding miscarriages.
Premature labor	A modification of the cervix associated with regular uterine contractions (ultrasound and fetal monitoring)
Hypertensive pathologies	Classified into three categories: chronic hypertension pre-existing before the pregnancy or before 20 WG; gravidic hypertension occurring after 20 WG without proteinuria (> = 140/90); and pre-eclampsia with gravidic hypertension with proteinuria (> = 300mg/24h) after 20 WG.
HELLP syndrome	Associated hemolysis, hepatic cytolysis and thrombocytopenia
Stillbirth	The birth of a dead infant who weighed >500 grams or at or after 22 WG.
Miscarriage	The birth of a dead fetus or dead embryo before the 22 WG
Small for gestational age infants	Birth weight below the 10^th^ percentile for gestational age (Hadlock).
Delivery hemorrhage	Loss of more than 500 ml of blood during delivery or immediately post-partum (within 5 days of delivery).
*in-utero* death	stopping of the fetal heart activity after 14 WG

### Statistical analysis

STATA 12.0 (STATA Corporation, College Station, Texas, USA) was used. Analyses included descriptive statistics for individual variables and hypothesis tests for each variable with the outcomes under investigation (Fisher's exact test, Wald's test and Pearson's chi^2^). Conditional Poisson regression was used to model multivariate associations, with each exposed woman (EG) and the three unexposed women (NEG) following it modeled as a matched stratum [13].

The statistical analysis was performed in 2 steps. For each outcome, univariate analysis selected the statistically significant explanatory variables. Explanatory variables were derived from sociodemographic and medical informations collected from each patient. The explanatory variables tested were chosen from the literature relative to the outcome.

In a second step, the multivariate model for each outcome was constructed with the selected explanatory variables, the main exposure variable (DENV infected) and adjustment variables. The adjustment factors corresponded to socioeconomic or medical factors that were not comparable between the 2 groups and could have confounded the relation. The final model was obtained using a backwards stepwise method.

The level of significance was 5%. Considering a study power of 80%, a 15% difference between both arms and an Exposed/Unexposed ratio (EG/NEG) of 1/3, the estimated sample size was 79 women in EG and 237 women NEG.

### Ethics statement

Written informed consent was provided and signed by all subjects before enrolment. For minors, written consent was signed by both the participant and her legal representative. The study was approved by the French regulatory authorities CCTIRS (Advisory Committee on Information Processing in Material Research in the Field of Health n°12323), the CNIL (National Commission on Informatics and Liberty; authorization DR-2012-585) and the CEEI (Inserm's ethics committee/Institutional Review Board n° 00003888).

## Results

Between June 2012 and June 2015, 363 pregnant women with a history of fever during the pregnancy were included in the study. The biological tests for dengue on inclusion confirmed 82 cases of acute dengue fever (22.6%). These 82 women were included in the exposed group (EG). Following the matching protocol, 290 pregnant women without fever during their pregnancy were included in the non exposed group (NEG). Blood samples were collected from women in the NEG gave upon inclusion and at delivery and tested for dengue. Overall, 71 women were excluded from the NEG and nine from the EG (see Fig 1 for exclusion criteria). For 39 women who should have been included in NEG, there was no sample taken at inclusion or/and at delivery (technical problem or omission). The EG analyzed was thus composed of 73 women and the NEG of 219 women.

### Quality of the matching process

There were no significant differences between the two groups for care center (Wald's test p = 0.72), place of residence (Wald's test p = 0.84) or inclusion term (Wald's test p = 0.67).

Indeed, the pregnancy of 35.6% (78/219) of the NEG and 42.5% (31/73) of the EG was mostly followed in the hospital; 28.8% (63/219) of the NEG and 21.9% (16/73) of the EG were mostly followed at a mother and child care center and 31.5% (69/219) of the NEG and 31.5% (23/219) of the EG had been followed mostly at a private-practice. The remaining 4.1% of the NEG and EG had a mixed follow up between the three types of structures.

The place of residence of 43.4% of NEG women (95/219) and 39.8% of EG women (29/73) was in the main city of French Guiana (Cayenne); 26.5% of NEG women (58/219) and 30.1% (22/73) of EG women lived in the central part of French Guiana and 30.1% of NEG women (66/219) and 30.1% of EG women (22/73) lived in western French Guiana.

Finally, 22.8% of NEG women (50/219) and 15.1% of EG women (11/73) were included in the study before 14 WG.

### Comparability of the groups

The quality of pregnancy follow-up did not differ significantly between groups: first ultrasound before 14 WG (Wald p = 0.76), supervision of pregnancy started before 14 WG (Wald p = 0.63) and total number of prenatal consultations (Wald p = 0.99).

Socio-demographic characteristics, residence and medical history, apart from dengue, did not differ significantly between the two groups (Table 3). The median maternal age was 28 years [IQR = 10] in NEG and 28 years [IQR = 10] in EG. The time spent travelling was 30 minutes [IQR = 50] in NEG and 20 minutes [IQR = 40] in EG. Obstetrical history did not differ significantly between the two groups apart from gravidity (Wald p = 0.028).

**Table 3:** Comparison of the socio-demographic data, residence, and medical and obstetrical histories of women in the exposed and non exposed groups.

Socio-demographic and general characteristics during pregnancy	NEG n/N(%)	EG n/N(%)	p(Wald)
Age at inclusion < 20 years	29/219(13.2)	12/73(16.4)	.528
Age at inclusion ≥ 35 years	42/219(19.2)	11/73(15.1)	.476
Multidimensional precariousness (EPICES)	85/174(48.8)	37/67(55.2)	.711
Having a complementary mutual insurance	158/181(87.3)	53/70(75.7)	.437
Having had contact with family in the past 6 months	158/181(87.3)	52/69(75.4)	.402
Average duration of standing up during the day			
Less than half of the time	72/180(40)	21/68(30.9)	.487
Over half of the time	108/180(60)	47/68(69.1)	
Median time spent travelling (by car or motorbike each day)			
< 20 minutes	66/145(45.5)	32/57(56.1)	.254
> 20 minutes	79/145(55.5)	25/57(43.9)	
Carrying heavy loads during pregnancy (>10kg)	66/181(36.5)	29/69(42.0)	.412
Anemia < = 11g/dl during pregnancy	134/216(62)	51/73(69.9)	.454
**Medical history**			
Hypertension	4/219(1.8)	1/73(1.4)	.797
Diabetes	0/219	2/73(2.7)	NA
Hemoglobinopathy	14/219(6.4)	3/73(4.1)	.487
Thrombo-embolism	4/219(1.8)	1/73(1.4)	.797
Auto-immune disease	0/219	2/73(2.7)	NA
**Habits**			
Active smoking during pregnancy	11/204(5.4)	5/66(7.6)	.565
Alcoholism	2/200(1)	2/64(3.1)	.272
**Gravidity upon inclusion**			
Primigravida	52/218(23.8)	29/73(39.7)	**.028**
**Obstetrical history**			
Premature labor	13/166(7.8)	4/44(9.1)	.366
Premature delivery	20/166(12.0)	5/44(11.4)	.927
Intra-uterine growth retardation	7/166(4.2)	4/44(9.1)	.121
Hypertension	12/166(7.2)	1/44(2.3)	.549
Pre-eclampsia	12/166(7.2)	3/44(6.8)	.636
Delivery hemorrhage	13/165(7.9)	3/44(6.8)	1
Abortion	53/166(31.9)	8/44(18.2)	.237
Medical pregnancy interruption	7/166(4.2)	2/44(4.5)	.890
Miscarriage	53/166(31.9)	18/44(40.9)	.345
Intra-uterine fetal death	9/163(5.5)	2/44(4.5)	.700
Caesarian section	28/166(17.0)	7/44(15.9)	.880

### Dengue fever in the exposed group

By definition, all 73 women of the EG developed symptomatic dengue fever during their pregnancy.

The symptoms occured after the 28th week of gestation in 55% of cases, between 14 and 28 WG in 30% of cases, and before 14 WG in 15% of cases. Molecular analyses of 68 of the sera revealed viral genomic RNA in 61 cases; 97% (59/61) were serotype DENV-2 and 3% (2/61) serotype DENV-4 (Table 4).

**Table 4:** Positive DENV diagnostic tests in the DENV infected group (N = 73).

Positive DENV diagnostic tests	n(%)
RT-PCR and NS1	45(62)
NS1	6(8)
RT-PCR	16(22)
Dengue IgM	6(8)

According to the 2009 WHO classification [7], 27% (20/73) of the EG had dengue fever with at least one clinical or biological warning sign (Table 5). In one case, dengue occured at 37 WG evolved towards severe dengue (group C) with liver enzyme elevation to over 1000 times the normal value, renal failure (with oliguria) and plasma leakage (peritoneal effusion) mimicking a HELLP-syndrome (no schizocytes in the blood smear). Two patients were given platelet-rich plasma transfusions.

**Table 5:** Warning signs observed in 20 pregnant women (27% of dengue cases) with dengue in the CMFdeng study.

Warning signs (WHO 2009)	n(%)
Pain or sensitivity upon abdominal palpation (without uterus contraction)	7(10)
Pleural effusion (confirmed by ultrasound scan)	2(3)
Peritoneal effusion (confirmed by ultrasound scan)	2(3)
Spontaneous mucous hemorrhage (gum bleeding, hemoptysis, epistaxis, metrorrhagia, cutaneo-mucous purpura)	8(11)
Hematocrit increase (> 20% difference between the reference value^1^ and the maximum value) associated with low platelet concentration (< 100 000/mm3)	7(10)

^1^ the reference value was the hematocrit value obtained from normal follow up, when the women was not ill

Based on the interpretation of the Panbio IgM/IgG tests performed on 47 samples, we concluded that 76.5% (36/47) of confirmed dengue cases corresponded to primary dengue infection. Secondary dengue was not significantly linked to dengue with warning signs (19% of primary dengue cases (7/36) and 27% of secondary dengue cases (3/11) presented with warning signs; Pearson chi^2^ p = 0.6).

### Description of delivery

Most deliveries were vaginal. There was an emergency caesarian section for 18.3% (40/219) of the NEG and 13.7% (10/73) of the EG, and elective caesarian for 5.4% (12/219) of the NEG and 5.5% (4/73) of the EG. The delivery mode did not differ significantly between groups (Wald's test p = 0.568).

Cardio-fetal rhythm anomalies were observed during routine monitoring for 34.4% (22/64) of the EG and 30.4% (65/214) of the NEG (Wald's test p = 0.718).

In the NEG, 43.4% (95/219) of the newborns were boys and in the EG, 43.1% (31/72) were boys (Wald's test p = 0.978). The median of birthweight at delivery was 3230 g [2920g-3500g] in NEG and 3200g [2795g-3605g] in EG. There were 217 livebirths in the NEG and 70 in the EG; the 1 minute APGAR score did not differ significantly between the two groups (APGAR score of 10 for 75.5% (163/216) of the NEG and 76.8% (53/69) of the EG; Wald's test p = 0.915). Transient oxygen therapy was administered at birth to 10% (7/70) of newborns in the EG and 5.1% (11/217) in the NEG (p = 0.181).

### Pathological events during the pregnancy

Exposure to dengue fever during pregnancy was not significantly associated with pathological events during pregnancy or during the perinatal period (Table 6).

**Table 6:** Comparison of pathological events during pregnancy and delivery between women exposed to dengue during pregnancy (EG) and women not exposed to dengue (NEG) during pregnancy (Univariate analysis).

Occurrence of	NEG n/N(%)	EG n/N(%)	CRR (95%CI)	p(Wald)
Premature labor	19/219(8.7)	11/73(15.1)	1.74(0.83–3.65)	0.145
Premature delivery	19/217(8.8)	3/70(4.3)	0.47(0.14–1.60)	0.229
Hypertension	10/219(4.6)	2/73(2.7)	0.6(0.13–2.74)	0.510
Pre-eclampsia	10/219(4.6)	0/73(0)	/	1
Premature rupture of membranes	6/219(2.7)	2/73(2.7)	1(0.20–4.95)	1
Small for gestational age infants	18/218(8.2)	7/72(9.7)	1.17(0.49–2.79)	0.729
Death *in utero*	2/219(0.9)	3/73(4.1)	**4.5(0.75–26.93)**	**0.099**
Delivery hemorrhage	12/219(5.5)	6/67(9.0)	1.8(0.65–4.95)	0.255
Caesarian section	52/219(23.7)	14/73(19.2)	0.81(0.45–1.46)	0.478
Emergency caesarian	40/219 (18.3)	10/73(13.7)	0.75(0.37–1.50)	0.416

CRR: crude relative risk

The 22 premature deliveries occurred between 27 WG and 37 WG. Nearly one in every three women who presented with premature labor delivered prematurely (7/22 with premature labor and premature delivery). Having contact with one's family (other than parents and children) in the previous 6 months (item of EPICES scale) was a protective factor for premature delivery with an adjusted relative risk (ARR) = 0.13 (95%CI[0.02–0.79]; Wald's test p = 0.027) adjusted for history of abortion (Wald's test p = 0.3), for primigravida (Wald's test p = 0.223) and dengue fever (Wald's test p = 0.511).

Having contacts with one's family (other than parents and children) in the previous 6 months was also a protective factor for premature labor, with an ARR = 0.06 (95%CI 0.01–0.63; Wald's test p = 0.018) adjusted for history of abortion (Wald's test p = 0.216), for primigravida (Wald's test p = 0.566) and dengue fever (Wald's test p = 0.179).

Twenty-two women presented with a hypertensive pathology during pregnancy: three had chronic hypertension, nine had gravidic hypertension without proteinuria, and 10 had preeclampsia; two of the 22 women presented a HELLP-syndrome and one presented a retro-placentar hematoma (all three in the NEG: in five of the 22 cases there was *in utero* growth retardation and in five cases premature birth). Gravid hypertension and preeclampsia were more frequent among women with a history of preeclampsia ARR = 11.1 (95%CI = 1.6–78.6; Wald's test p = 0.016) and those aged 35 or more ARR = 7.5(95%CI = 1.4–39; Wald's test p = 0.016). Adjustment were made for primigravida (Wald's test p = 0.098), history of abortion (Wald's test p = 0.281) and dengue fever (Wald's test p = 0.303).

Eighteen women had a delivery hemorrhage, including nine who lost at least 800 ml of blood (min/max: 500/1500ml); 11/18 had a uterine revision. Two women having had caesarian section had a major hematoma in the immediate post-partum period and one woman had abundant peroperative bleeding. One woman with a parietal hematoma was given a blood transfusion.

Of 18 deliveries, 17 newborns were alive at birth. The median birth weight was 3162g (2880–3910) with one small for gestational age child. Fifty-three percent of newborns (9/17) had an APGAR score of 10 at 1 minute of life and one child required respiratory support at birth.

In the EG, 18.2% (4/22) of the women having presented dengue close to delivery (within 15 days before or 4 days after delivery) and 4.4% (2/45) having had dengue earlier before term presented abundant bleeding during delivery or the immediate post-partum (Fisher's exact test: Wald's test p = 0.08).

Having had dengue with at least one WHO warning sign was a risk factor for hemorrhage during labor or the immediate post-partum period with a crude relative risk (CRR) of 6 (95%CI = 1.1–32.7) and an ARR of 8.6 (95%CI = 1.2–62) after adjusting for primigravida and history of abortion (Table 7). The attributable risk for exposure to dengue and severe bleeding was 31% with a frequency of exposure of 6% (18/286).

**Table 7:** Univariate and multivariate analysis of risk factors for hemorrhage during delivery or the immediate post-partum (Conditional Poisson regression).

			univariate analysis		multivariate analysis	
Occurrence of delivery hemorrhage among women:	Yes n(%)	No n(%)	p	CRR (95%CI)	p	ARR (95%CI)
Without dengue	12(66.7)	207(77.2)		1		1
Uncomplicated dengue	2(11.1)	47(17.5)	0.716	0.7(0.2–3.5)	0.821	0.8(0.2–4)
**Dengue with warning signs**	4(22.2)	14(5.2)	**0.039**	**6(1.1–32.7)**	**0.033**	**8.6(1.2–62)**
*Primigravida*	5(27.8)	71(26.6)	0.656		0.248	
History of abortion	6(33.3))	55(20.6)	0.288		0.165	
Anemia during pregnancy (<11g/dl)	16(88.9)	168(63.4)	0.059			
Caesarian section	5(27.8)	61(22.7)	0.614			
History of delivery hemorrhage	2(11.1)	14(5.3)	0.272			

CRR: crude relative risk

ARR: adjusted relative risk

A history of a caesarian section (ARR = 3[1.4–6.4]; Wald's test p = 0.005) and cardio-fetal rhythm anomalies during labor (ARR = 2.1[1.1–3.9]; Wald's test p = 0.027) were associated with an increased risk of caesarian section after adjustment for dengue (Wald's test p = 0.545), history of abortion (Wald's test p = 0.425) and being primigravida (Wald's test p = 0.257).

Concerning the two *in utero* deaths in the NEG occurred at 34 WG and 39 WG, in the context of retro-placentar hematoma and preeclampsia. In the EG, there was an interrupted pregnancy at 11 WG and two fetal deaths *in utero* at 25 and 33 WG; RT-PCR tests of samples of placenta for dengue viral RNA were negative for all three cases. There was no factor significantly linked to death *in utero*. However, the association between having had dengue and death *in utero* was close to statistical significance (Wald's test p = 0.09). Two of dengue cases with warning signs (n = 20) and one of dengue cases without warning signs (n = 53) were *in utero* deaths.

## Discussion

This prospective cohort of dengue cases during pregnancy has three features of particular note.

The first is the criteria of exposure and non exposure. The women included in the exposed group (EG) presented with biologically confirmed dengue fever; in 92% of cases it was confirmed by detection of viral RNA and/or of NS1 antigen, both very specific techniques for confirming the diagnosis and the acute nature of the infection [14]. The confirmation of the diagnosis by serology (detection of dengue IgM) for other women (8% of the EG group), did not allow to date the infection with certainty (between 7 days and 6 months) [15]. However, women past the sixth month of pregnancy could be included in the EG on the basis of positive IgM tests. The centralized analysis of samples in the same laboratory minimized bias associated with variability between laboratories using different protocols or reagents. Women in the NEG group were only included in the analysis if they had negative diagnostic tests on samples collected both at inclusion and at the end of the study. This avoided the risk of including women having presented symptomatic or asymptomatic dengue between inclusion and delivery in the NEG group.

The second feature is that factors that could bias the measurement of events during follow up were considered. Matching limited social differences linked to the place of follow up and avoided recruitment being disproportionately in hospitals. Matching on the gestational week at inclusion limited the underestimation of events at the beginning of pregnancy in the NEG group: if the NEG was recruited at the end of pregnancy only, pathological events during the first trimester (miscarriages for example) would be missed. Socio demographic factors and medical history collected for all women were analyzed. There were no significant differences between the two groups for these factors, except for gravidity (more EG women were primigravida and more NEG women were multigravida). Gravidity was therefore added to the adjustment variables in the multivariate analysis in order to control for their potential confounding effect.

Finally, the third feature is that the study was prospective in design, as is appropriate for the study aims (outcome prevalence study) [16]. To compensate for the small sample size of the EG (expected number <100 over 3 years) and to maximize the power of the study, three controls were included for every case [17]. Although there was no randomization or double blinding, the pregnancy follow up (number of consultations and term at the beginning of follow up) were not significantly different between the two groups.

The first finding of the study is that, without adjusting for potential confounders, exposure to dengue fever (without distinction between dengue with and without warning signs) did not significantly affect either pathological events or outcomes of pregnancy.

The EPICES score indicated that nearly half of the women sampled (48% of the NEG and 52% of the EG) were socially vulnerable. A precarious situation may be associated with delayed and/or poor pregnancy follow up [12]. Isolation from the family (moral support, material help) increased the risk of each premature labor and premature delivery. This confirms the relevance of socio economic conditions to the complications of pregnancy and both analysis and comparative studies [18].

According to a recent review, the premature delivery rate for women with dengue was 4% among the 25 births reported in case reports and 16.1% among the 143 births reported in cases series [1]. In a meta-analysis of five studies, the odds ratio for the association between dengue during pregnancy and preterm birth was 1.71 [19]. In our study, the rate was 4.3% in the EG and 8.8% in the NEG. A previous retrospective comparative study in French Guiana, found that 17.4% of births to women with dengue and 8.7% of births to mothers without dengue were before 37 WG[4]; the risk of premature delivery was thus higher for women with symptomatic dengue with an OR of 3.34 (1.13–9.89) adjusting for gravidity but not for obstetrical history. The non exposed group in this retrospective study included women chosen retrospectively without biological confirmation of their non exposure to dengue, and prematurity included miscarriages. In our study, premature delivery and labor were best explained by socio economic conditions, whether the women had dengue during pregnancy or not.

We found no evidence that the risk of preeclampsia and caesarian section were influenced by exposure to dengue fever; this risk was influenced by obstetrical history. The few cases of preeclampsia observed in pregnant women with dengue may have been the consequence of excessive fluid replacement, or preexisting hypertension, or a history of preeclampsia [20–22].

The definitions used for hypotrophy differ between studies. Most studies defined low birth weight (LBW) as a birth weight of a live-born infant of less than 2,500 g regardless of gestational age [4,23–25].However, prematurity alone may explain LBW; a birth weight below 2,500 g may be normal for the term [1]. A comparative study reported that 4/22 births to dengue infected mothers were LBW whereas there were 0/24 cases in the group not exposed to dengue; however, three of the four newborns with LBW were born prematurely [23].

In our study, pregnant women with dengue with warning signs had a 8.6 times greater risk of severe bleeding during delivery or *perpartum* than women who did not have dengue. According to the attributable risk, nearly one in three (31%) severe bleeding cases is attributable to the exposure to dengue with warning signs during pregnancy. In Mexico, a retrospective analysis also highlighted that severe dengue was associated with obstetrical hemorrhage. In this study among 82 dengue cases, obstetrical hemorrhage occurred in five patients: four (30.8%) with severe dengue and one (1.9%) nonsevere dengue [26]. Warning signs during the disease indicate a critical phase and mostly result from capillary leakage. Hemorrhagic signs may complicate this phase (rapid onset of hemoconcentration and thrombocytopenia) [7]. The hemorrhagic risk did not only seem related to the severity of thrombocytopenia. Severe bleeding in the EG was not necessarily linked to maternal dengue close to term. Because of the risk of bleeding during surgical procedures, some authors recommend vaginal delivery for women with dengue [21,27,28]. In our study, the mode of delivery was not linked to the occurrence of severe bleeding.

The association between dengue and *in utero* death (miscarriages and stillbirth) was close to statistical significance (10% of exposed women with warning signs versus 1.9% of unexposed women). It is likely that this association is genuine, but that our study was not sufficiently powerful to reach statistical significance. Cases of *in utero* death associated with dengue and in particular after hemorrhagic dengue have been reported [3,29]. A comparative study in Malaysia reported that recent maternal dengue was associated with an increased risk of miscarriage up to 22 WG [30].

Viral infection may cause fetal death through various mechanisms including direct infection, placental damage or severe maternal illness [31,32]. Nearly one in five fetal deaths before 28 WG are associated with an infection, and 2% of term stillbirths are infection-related [33].

The fact that nearly half of DENV infections occurred before 28 weeks gestation may have affected the observation of late pregnancy or delivery complications, which could only appear when dengue occurs after 28 weeks.

## Conclusion

We studied the consequences of symptomatic dengue in pregnant women by applying a matched cohort methodology. We report two main results: i) controlling for confounding socio-economic and obstetrical events is crucial for the analysis of the outcomes of dengue during pregnancy; and ii) severe manifestations in the mother are associated with poor outcomes notably *peripartum* hemorrhage [19,34]. These results indicate that adapted management of symptomatic dengue in pregnant women would be beneficial [7]. Management should involve looking for warning signs, repeated ultrasound monitoring of fetal vital signs, and preventive and curative treatments against *peripartum* hemorrhage [33].

Complementary studies of severe bleeding during delivery and death *in utero* are required to elucidate the pathogenesis of poor obstetrical outcomes associated with maternal dengue. It would also be valuable to conduct other larger studies controlling for confounders in other dengue-endemic regions to determine whether these results in French Guiana can be replicated and refined beyond our study setting [19].
